# Cattle Immunization with T7 Phage-Displayed Whole-Tick Antigens Reduces *Amblyomma americanum* Feeding Efficiency and Blocks Larval Tick Hatching

**DOI:** 10.3390/pathogens15030281

**Published:** 2026-03-05

**Authors:** Moiz Ashraf Ansari, Alex Kiarie Gaithuma, Thu-Thuy Nguyen, William Tae Heung Kim, Emily Bencosme-Cuevas, Jacquie Berry, Jennifer Fridley, Kimberly Lohmeyer, Marie-Eve Koziol, Albert Mulenga

**Affiliations:** 1 Department of Veterinary Pathobiology, School of Veterinary Medicine and Biomedical Sciences, Texas A&M University, College Station, TX 77843, USA; moiz@tamu.edu (M.A.A.); akiariegaithuma@gmail.com (A.K.G.); ttnguyen@cvm.tamu.edu (T.-T.N.); wkim@cvm.tamu.edu (W.T.H.K.); emily.bencosmecuevas@nih.gov (E.B.-C.); jberry@cvm.tamu.edu (J.B.); 2 Molecular Parasitology & Entomology Unit, Laboratory of Malaria & Vector Research, National Institute of Allergy and Infectious Diseases (NIAID), National Institutes of Health (NIH-NIAID), Rockville, MD 20852, USA; 3 Veterinary Large Animal Clinical Sciences, Veterinary Medical Park, Texas A&M University, College Station, TX 77843, USA; jfridley@cvm.tamu.edu; 4 Knipling-Bushland U.S. Livestock Insects Research Laboratory, Agricultural Research Services, United States Department of Agriculture (USDA-ARS), Kerrville, TX 78028, USA; kim.lohmeyer@usda.gov; 5 Adjuvants Division, SEPPIC Inc., Fairfield, NJ 07004, USA; marie-eve.koziol@airliquide.com

**Keywords:** ticks, tick-borne diseases, tick vaccines, immunizing cattle against tick feeding, tick vaccine antigens, T7 phage display system

## Abstract

This study demonstrates the feasibility of using a T7 phage display platform to deliver a library of tick antigens as a vaccine to disrupt tick feeding in cattle. Cattle were vaccinated at three-week intervals via intradermal and intramuscular routes with a cocktail of male and female *Amblyomma americanum* T7 phage display cDNA libraries, with and without adjuvant. ELISA and Western blot analyses confirmed that vaccinated cattle mounted immune responses directed against phage-displayed tick proteins rather than the T7 phage backbone. Vaccine-induced antibodies recognized both native tick salivary gland proteins and selected recombinant salivary proteins, indicating effective antigen presentation and biologically relevant immunity with binding to native tick saliva proteins. The adjuvanted formulation elicited significantly stronger immune responses than phage-only immunization. Immunized cattle exhibited robust immune memory, evidenced by a pronounced anamnestic response following tick infestation. This immunity translated into measurable anti-tick effects, including reduced tick feeding efficiency and blood ingestion. Tick reproductive success was severely compromised, with larval hatching declining from 54% in ticks fed on control cattle to 4% in ticks fed on immunized cattle. This study establishes a practical and scalable T7 phage-displayed whole-tick antigen platform capable of inducing durable anti-tick immunity in cattle.

## 1. Introduction

Ticks and tick-borne diseases pose significant challenges to both public and veterinary health. In tropical regions, they remain a leading cause of economic loss in the livestock industry. Recent estimates place the annual global loss caused by ticks and tick-borne diseases in livestock between $13.9 billion and $18.7 billion [1,2]. Globally, ticks rank second only to mosquitoes in terms of their impact as vectors of human disease. However, in the United States, ticks are the leading vectors of most human vector-borne disease pathogens [1,2,3]. Tick-borne illnesses, such as Lyme disease, are estimated to result in annual economic losses of approximately $1.3 billion [2,3].

There are currently no effective vaccines against most tick-borne disease pathogens, and prevention efforts depend on the use of acaricides to kill ticks. However, the widespread emergence of acaricide-resistant tick populations [4,5] threatens the long-term effectiveness of this approach. This challenge underscores the urgent need for alternative tick control strategies. Among these, immunizing animals to block tick feeding has emerged as the most sustainable and promising method [6].

The concept of anti-tick immunization originated from observations that repeated tick infestations can induce strong immune responses that significantly impair subsequent tick feeding and transmission of tick-borne diseases [7,8]. To replicate this naturally acquired immunity, several strategies have been explored, including immunization with crude native tick protein extracts [9] and recombinant tick proteins [9,10]. The successful commercialization of a vaccine targeting *Rhipicephalus microplus* based on a tick midgut protein, Bm86 [11] validated the potential of anti-tick immunization. However, the inconsistent efficacy of the Bm86 vaccine has highlighted the need to develop more reliable and broadly effective alternatives.

Our laboratory focuses on identifying key tick saliva proteins that regulate tick feeding and pathogen transmission and exploring how these proteins can be targeted in vaccines to block both processes [12,13]. Work from our group and others indicates that the immunity observed in repeatedly infested animals is driven by multiple, yet largely unidentified, tick saliva proteins [14]. Through a series of studies, we have identified saliva proteins that are injected into the host to facilitate feeding, many of which represent promising targets for vaccine development [15]. Notably, some of these proteins are secreted early in the feeding process, making them ideal candidates for disrupting pathogen transmission [15]. Studies have consistently shown that immunizing animals with complex salivary gland extracts, which contain multiple proteins, induces potent protective immunity against tick feeding [16,17]. These findings strongly support the notion that a multicomponent vaccine comprising a cocktail of tick saliva proteins can confer robust protection.

Motivated by published findings that bacteriophage-displayed antigens can induce specific immune responses [18], we initiated this study to evaluate whether immunizing cattle with a cDNA expression library cloned in the T7 phage display system could elicit protective immunity against ticks. A key advantage of the T7 phage platform is its ability to present foreign antigens on the phage capsid surface, enabling direct interaction with the host immune system [19,20]. Importantly, although T7 phages can enter mammalian cells, they do not replicate within them [21]. Recent studies also demonstrate the successful use of phage display systems to develop vaccines against other vector-borne diseases, further supporting this approach [18,22]. We previously characterized T7 phage display cDNA expression libraries of male and female *A. americanum* ticks [15]. In this study, we show that immunization of cattle with the *A. americanum* T7 phage cDNA expression libraries elicits specific anti-tick immune responses that impair tick feeding efficiency and inhibit larval tick hatching. These results provide a promising foundation for developing multicomponent phage-based vaccines that target multiple tick saliva proteins, thereby mimicking the broad immunity acquired through repeated tick infestations.

## 2. Methods

### 2.1. Ethics Statement and Sources of Cattle and Ticks

The described experimental procedures were executed following the animal use protocol approved by Texas A&M University Institutional Animal Care and Use Committee (IACUC#s 2020-0094 and 2023-0093) to meet all federal requirements, as defined in the Animal Welfare Act (AWA), the Public Health Service Policy (PHS), and the Humane Care and Use of Laboratory Animals.

For the overall study, a total of ten age-matched male Holstein steer calves, aged 19–21 weeks at the beginning of the study, were sourced from Hanna Dairy in Godley, TX, USA. Ticks used in this study were either purchased from the Oklahoma State tick laboratory, Stillwater, OK, USA, or were kindly provided by our collaborators at the USDA-ARS in Kerrville, TX, USA. Routinely, unfed and fed ticks were kept in a secure plastic container at room temperature, with relative humidity maintained at >85%.

### 2.2. Preparing the T7 Phage Display Library Inoculum

In a previous study, we constructed T7 phage display cDNA expression libraries from *A. americanum* males and females that had fed on rabbits for 24 and 96 h, respectively [15]. Male and female tick phage display libraries were amplified separately as described previously [15]. In this study, the prepared library was purified via glycerol gradient ultracentrifugation as described elsewhere with some modifications [23]. Initially, phage particles were precipitated by adding 20% PEG-8000 and 2.5 M NaCl to the solution, followed by incubation on ice at 4 °C overnight. The precipitate was recovered by centrifugation at 8000× *g* for 10 min at 4 °C, and the supernatant was discarded. The pellet was resuspended in 1.5 mL STE buffer (1 M NaCl, 10 mM Tris-HCl, pH 8.0, 1 mM EDTA) and vortexed for 30 s. For gradient purification, 5% and 40% glycerol solutions were prepared in SM buffer (50 mM Tris-HCl pH 7.5, 100 mM NaCl, 8 mM MgSO_4_). A glycerol gradient was formed by layering 17 mL of 40% solution with 5% solution on top. The phage sample was carefully layered on the gradient, and tubes (Beckman Coulter, Brea, CA, USA) were balanced and centrifuged at 165,000× *g* for 90 min at 4 °C. After centrifugation, the gradient layers were removed, and the phage pellet was resuspended in SM buffer (200 mM NaCl, 10 mM MgSO_4_, 50 mM Tris-HCl, pH 7.5). Endotoxin removal was performed by adding 1% Triton X-114 to the phage suspension, followed by vortexing, incubation on ice for 15 min, a second vortex, and centrifugation at 15,000× *g* for 1 min at 37 °C. The upper aqueous phase containing purified phages was collected for downstream applications, including immunization. Each purified phage display library (male and female tick) was quantified by plaque-forming units (PFU) assay. Equal amounts of each library were combined to create the *A. americanum* male and female tick T7 phage display library cocktail, which was used to immunize cattle.

### 2.3. Cattle Immunization

Vaccine preparations were made using All-Plastic Syringes (Henke Sass Wolf GmbH, Tuttlingen, Germany). One syringe was loaded with 1.5 × 10^14^ PFU of the *A. americanum* T7 phage display library (or SM buffer for controls), and the other with Montanide™ ISA 61 VG adjuvant, a water-in-oil formulation (Seppic Inc., Fairfield, NJ, USA). The syringes were connected using a double female Luer lock connector (Smiths Medical ASD, Inc., Dublin, OH, USA) and mixed by transferring the contents back and forth for 20 slow and 60 rapid cycles. Emulsion stability was confirmed using the drop test: an emulsion drop that remained intact on water overnight at room temperature was considered stable. Final vaccine formulations consisted of 800 µL of phage preparation (1.9 × 10^14^ PFU in SM buffer) mixed with 1.2 mL of adjuvant to a total volume of 2 mL. Control animals received 800 µL of SM buffer mixed with 1.2 mL of adjuvant.

Cattle immunization studies were conducted in two phases (Rounds 1 and 2). The first study (Round 1) evaluated the effects of immunizing cattle, with the *A. americanum* T7 phage display library cocktail administered either with or without adjuvant. Outcomes from the first study suggested that adding an adjuvant enhanced efficacy, and thus the study was repeated to validate these findings. In the first study, six age-matched, male Holstein steer calves (Hanna Dairy, Godley, TX, USA) with no prior tick exposure were randomly assigned into three groups of two. Group 1 (control) received intramuscular (IM) and intradermal (ID) injections of adjuvant mixed with SM buffer only. Groups 2 and 3 received the *A. americanum* T7 phage display library cocktail mixed with either adjuvant (Group 2) or SM buffer without adjuvant (Group 3). Both priming and booster doses were administered three weeks apart at five sites near the neck (IM and ID), with 200 µL injected per site. The total dose (200 µL per site across five sites) was administered via intramuscular (IM) and intradermal (ID) routes, and the distribution and injection pattern were kept consistent for all animals across all groups to ensure experimental reproducibility.

**Figure 1:**
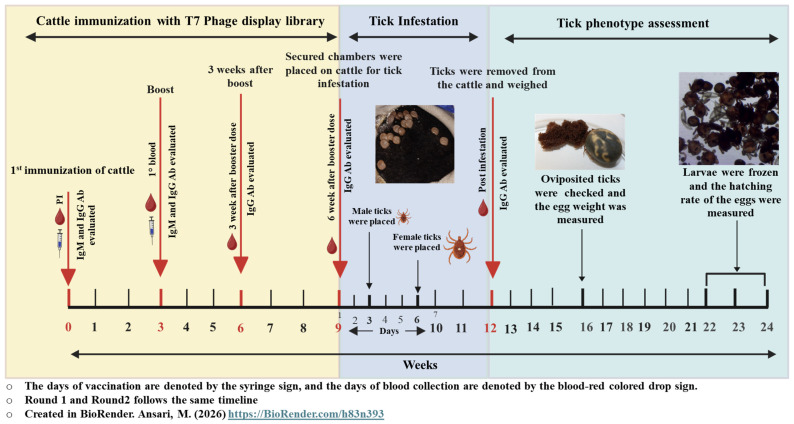
Timeline of experiments. The timeline represents weeks, counted from the first immunization. The experimental timeline is broadly divided into three parts: cattle immunization (yellow box), tick infestation (blue box), and tick phenotypic studies (green box). In the yellow and blue boxes, a drop sign represents the days on which blood is drawn. The weeks of vaccination are denoted by the syringes drawn beside the arrows, which were done at 3-week intervals using AaT7Փ cocktail and adjuvant emulsion. The Green box shows the weeks during which the phenotype, such as engorgement weight, egg weight, and the rate of hatching, was observed.

In the second study (Round 2) to validate the first-round findings, four new cattle were equally divided into two groups. One group received the adjuvant-only control, and the other received the *A. americanum* T7 phage display library cocktail with adjuvant formulation, prepared as described above. Blood samples for serum isolation were routinely collected from the jugular vein as indicated in the workflow (Figure 1).

### 2.4. Immunoglobulin (Ig) G Purification from the Cattle Serum

To precisely compare immune responses between groups, IgG was isolated from sera of all immunization stages using Rockland Sepharose™ Protein A/G beads (Rockland Immunochemicals, Inc., Limerick, PA, USA), following the manufacturer’s instructions with slight modifications. Briefly, cattle serum was clarified by centrifugation at 10,000× *g* for 10 min to remove particulates. The clarified serum was diluted 1:1 with phosphate-buffered saline (PBS) and incubated with pre-equilibrated Protein A/G beads at 4 °C for 2 h with gentle rocking to facilitate IgG binding. Following incubation, the bead-serum mixture was transferred into a chromatography column (Bio-Rad, Hercules, CA, USA) and allowed to settle for 30 min. Air bubbles were removed by gentle tapping, and unbound serum was drained from the column. The beads were then washed three times with PBS to remove unbound proteins. IgG was eluted using 0.1 M citric acid (pH 3.0), and eluted fractions were immediately neutralized with 1 M Tris-HCl (pH 9.0) to prevent protein denaturation. Purified IgG fractions were collected, dialyzed in PBS, and stored at −80 °C. The concentration was determined using the standard BCA protein assay (Thermo Fisher Scientific, Waltham, MA, USA), and the quality of the purified IgG was assessed with silver staining and Western blotting.

### 2.5. ELISA to Validate Immunogenicity

To assess the immunogenicity of T7 phage-displayed *A. americanum* whole-tick proteins, purified phages, native protein extracts from dissected salivary glands of *A. americanum* ticks fed on cattle for 24, 48, 72, and 96 h, and recombinant tick saliva proteins were subjected to standard ELISA analysis using tick immune antibodies. The use of tick salivary gland protein extracts was based on the rationale that salivary gland-expressed mRNAs encode for proteins that are injected into the host during tick feeding. The recombinant saliva proteins were selected based on prior evidence of their injection into the host during tick feeding [15,24] and their identification in the *A. americanum* immuno-proteome [15]. Tick salivary glands were dissected following established protocols [25], while recombinant proteins were expressed in *Pichia pastoris* and purified via affinity chromatography using laboratory-established methods [12,13].

To evaluate antibody responses against T7 phage-displayed antigens, Nunc Polysorp 96-well plates (Thermo Scientific, Rochester, NY, USA) were coated with the *A. americanum* T7 phage display cocktail, starting at 1 × 10^10^ PFU and twofold serially diluted across 10 wells in carbonate-bicarbonate coating buffer (4 mM Na_2_CO_3_, 9 mM NaHCO_3_, pH 9.4). Plates were incubated overnight at 4 °C, washed three times with PBS containing 0.05% Tween-20, and blocked with 3% BSA (200 µL/well) for 2 h at room temperature.

For IgM detection, cattle serum samples were diluted 1:500 in 1% BSA and added to the antigen-coated wells (100 µL/well). Plates were incubated for 4 h at room temperature. After six washes, HRP-conjugated sheep anti-bovine IgM (1:2000; Fortis Life Sciences, Boston, MA, USA) diluted in blocking buffer was added (100 µL/well) and incubated for 1 h at room temperature. For IgG detection, plates were incubated with affinity-purified bovine IgG (25 µg/mL; 100 µL/well). After six washes, HRP-conjugated chicken anti-bovine IgG (H + L) (1:2000; Thermo Fisher Scientific, Waltham, MA, USA) diluted in blocking buffer was applied (100 µL/well) and incubated for 1 h at room temperature. Plates were developed using 1-Step Ultra TMB-ELISA Substrate Solution (Thermo Fisher Scientific, Waltham, MA, USA); the reaction was stopped with 2 N H_2_SO_4_, and optical density was measured at 450 nm using a microplate reader (BioTek Instruments Inc., Winooski, VT, USA).

To confirm the specificity of the immune response to native tick saliva proteins, ELISA plates were coated with tick salivary gland protein extracts (100 ng/well) and processed for IgG binding as described above. To assess the immune response to specific recombinant proteins, IgG antibody responses to affinity-purified recombinant *A. americanum* saliva proteins (100 ng/per well) were evaluated as outlined above.

### 2.6. Western Blotting Analysis to Validate Specific Immunogenicity

Protein extracts (3 µg) of dissected tick salivary gland described above, or affinity-purified recombinant tick saliva proteins (1 µg, Table 1), were resolved on SDS-PAGE on 12% polyacrylamide gels and subjected to standard Western blotting. Membranes were blocked in 5% skim milk in PBS-T for 2 h at room temperature to prevent non-specific binding. Following blocking, membranes were incubated overnight at 4 °C with purified IgG (25 µg/mL). Subsequently, membranes were washed three times with PBS containing 0.1% Tween-20 (PBS-T), and then incubated with a 1:5000 dilution of HRP-conjugated chicken anti-bovine IgG (H + L) antibody (Life Technologies, Frederick, MD, USA) for 1 h at room temperature. Membranes were subsequently washed five times with PBS-T, developed using SuperSignal™ West Femto substrate (Thermo Scientific, Houston, TX, USA), and visualized using a Bio-Rad chemiluminescence imaging system.

### 2.7. Tick Challenge

Two weeks after the booster dose, cattle were challenged with adult *A. americanum* ticks as previously described [15]. A twenty-inch cotton stockinette sleeve tick containment patch was secured to each calf using Kamar^®^ adhesive (Kamar Inc. Zionsville, IN, USA). To ensure that the male ticks were primed to inseminate females, they were placed on cattle three days before the addition of female ticks. Each patch was initially infested with 15 adult male ticks; then, after three days, 20 adult female ticks were introduced into the patch. The feeding patches were inspected daily for their proper attachment, but were opened every other day to monitor the progression of tick attachment and feeding. Once replete, females began to detach, and the patches were opened daily to collect all engorged ticks that had self-detached. This process was repeated daily until all ticks had completed feeding. After the tick completed feeding, the patches were removed using an adhesive remover suitable for use on livestock (Weaver Leather LLC, Millersburg, OH, USA). Then the patched and the tick-infested area were cleaned with 2% Chlorhexidine solution (Durvet, Inc., Blue Springs, MO, USA) to reduce any potential infection on the wounds of the tick bite.

### 2.8. Evaluating the Effects of Immunization on Tick Feeding and Reproduction

The impact of immunization on tick feeding and reproductive efficiency was assessed as previously described [15]. To evaluate feeding success, the following parameters were measured: female tick attachment rates, time to complete feeding, and engorgement weight as a proxy for the amount of ingested blood. Reproductive efficiency was evaluated based on egg mass weight, egg mass conversion ratio, and larval hatching rates. After detachment, engorged female ticks were sequentially washed with 70% ethanol and sterile water, weighed, and individually placed in Petri dishes (VWR, Missouri, TX, USA) lined with moist filter paper to maintain humidity. The dishes were sealed with parafilm (Parafilm, Neenah, WI, USA) to prevent desiccation and incubated at 25 °C for egg laying. Ticks were monitored daily for visible morphological changes and egg laying. Following oviposition, egg masses were weighed, and the egg mass conversion ratio (i.e., the proportion of blood converted into eggs) was calculated using the formula: (Egg mass weight/Engorgement weight) × 100. To assess egg hatching rates, eggs were incubated at 25 °C with 85% relative humidity, and upon hatching after 4 weeks, larvae and unhatched eggs were frozen at −80 °C to kill hatched larvae. Subsequently, hatched larvae and unhatched eggs were manually counted. The hatching rate was determined by calculating the ratio of larvae to total eggs using the formula: % Larvae Hatched = (Number of larvae/[Number of larvae + unhatched eggs]) × 100.

### 2.9. Statistical Analysis

Statistical analyses were conducted using GraphPad Prism version 10 (GraphPad Software Inc., La Jolla, CA, USA). Unless otherwise stated, data are presented as mean ± standard error of the mean (SEM). The statistical analysis for ELISA was measured by Student’s *t*-test, and the group comparisons for the statistical analysis of tick feeding and reproduction efficiency were performed using unpaired *t*-tests with Welch’s correction to account for unequal variances. A *p*-value of <0.05 was considered statistically significant. Graphs and data visualizations were generated using GraphPad Prism 10 and Microsoft Excel.

## 3. Results

### 3.1. Immunization of Cattle with T7 Phage-Displayed A. americanum Whole-Tick Antigens Elicited Specific Antibodies Against Phage-Displayed Tick Antigens and Native Tick Salivary Gland Proteins

**Figure 2:**
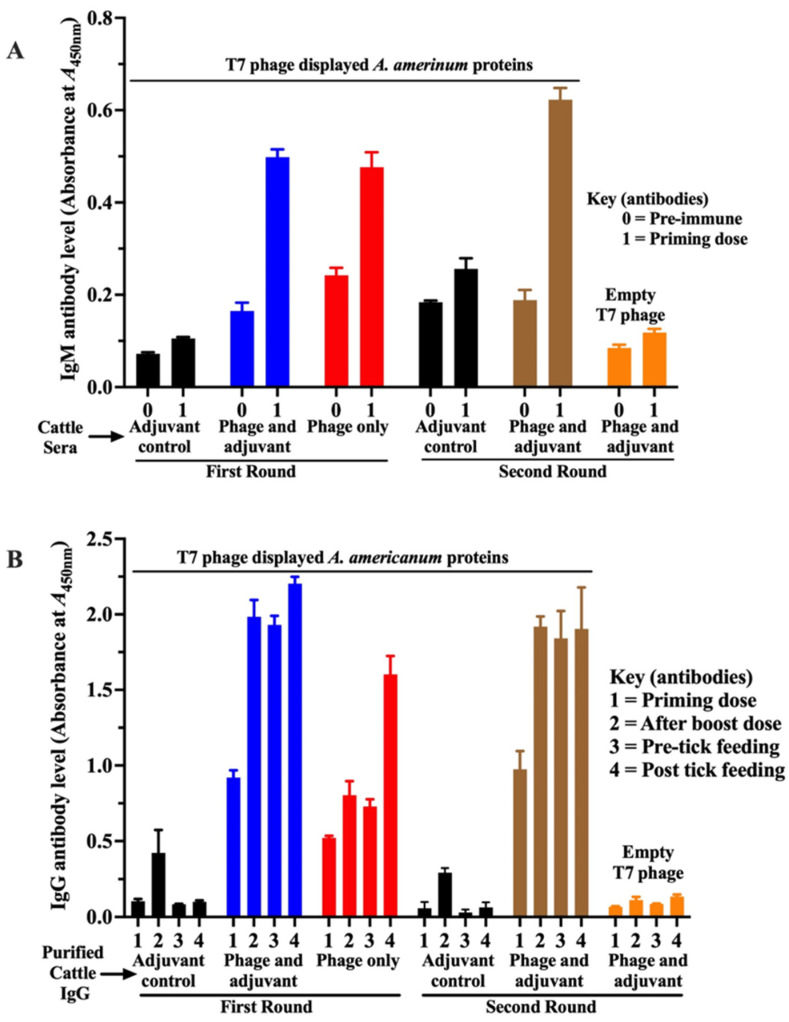
Immunization of cattle with T7 phage-displayed *Amblyomma americanum* whole-tick antigens induces antigen-specific IgM and IgG antibody responses. T7 phage display library expressing *A. americanum* whole antigens (10^10^ plaque-forming units) was coated on ELISA plates and screened for IgM and IgG antibody binding using standard ELISA. (**A**) IgM antibody response to T7 phage-displayed antigens. Levels in cattle sera were measured before immunization (0) and three weeks after the priming dose (1). ELISA screening included sera from control cattle (SM buffer + adjuvant, black bars), T7 phage display library + adjuvant (blue bars), and T7 phage only (red bars) from the first round of immunization. In second round, screening included adjuvant control (black bars) and T7 phage display library + adjuvant treatment (brown bars). Empty T7 phage control (orange bar) for negative control was screened using serum from T7 phage display library + adjuvant-immunized cattle. The *Y*-axis shows mean optical density (OD) values at A450nm with standard error of mean (SEM). (**B**) IgG antibody response to T7 phage-displayed antigens using purified IgG. Levels were assayed in cattle immunized with T7 phage-displayed antigens formulated with or without adjuvant. IgG was purified from cattle sera at three weeks after the priming dose (bar 1); three weeks after the booster dose (bar 2), before tick feeding (bar 3), and at one day after tick feeding (bar 4). The *Y*-axis shows mean A450nm values with SEM. Statistical significance between immunized and control groups was determined using Student’s *t*-test.

We first assessed whether the immune response elicited by *A. americanum* T7 phage-displayed proteins was specific to the T7 phage-displayed tick antigens rather than the phage itself. As shown (Figure 2), immunization with the T7 phage-displayed tick antigen cocktail elicited a specific immune response against the T7 phage-displayed tick proteins. Since the primary immune response begins with IgM production followed by class switching to IgG, we first measured IgM levels three weeks after the priming dose, but prior to the booster immunization (Figure 2). ELISA analysis using the T7 phage-displayed tick antigen cocktail (1 × 10^10^ PFU/mL) revealed significantly elevated IgM levels (more than four-fold higher, *p* value ≤ 0.0001) in cattle immunized with either the T7 phage-displayed tick antigen cocktail alone or the cocktail formulated with adjuvant, compared to control cattle immunized with adjuvant alone (Figure 2A). In contrast, IgM reactivity to empty phage was comparable to background levels in the adjuvant controls, confirming the specificity of the response to the tick proteins displayed on the T7 phage (Figure 2A).

We next evaluated IgG responses to T7 phage-displayed antigen immunization (Figure 2B). Consistently, cattle immunized with T7 phage-displayed tick antigens developed strong IgG responses to T7 phage-displayed proteins compared to the adjuvant-only inoculated control or against empty phage particles. Notably, formulating the immunogen with an adjuvant was essential for achieving high IgG titers. Although IgG levels were similar across groups at the priming dose, post-boost and pre-infestation sera of immunized cattle had significantly elevated IgG levels (*p* value ≤ 0.0001), which were approximately two-fold higher than those in cattle immunized without adjuvant formulation. Notably, these observations were reproduced in our second-round immunization with adjuvant-formulated T7 phage-displayed antigens.

**Figure 3:**
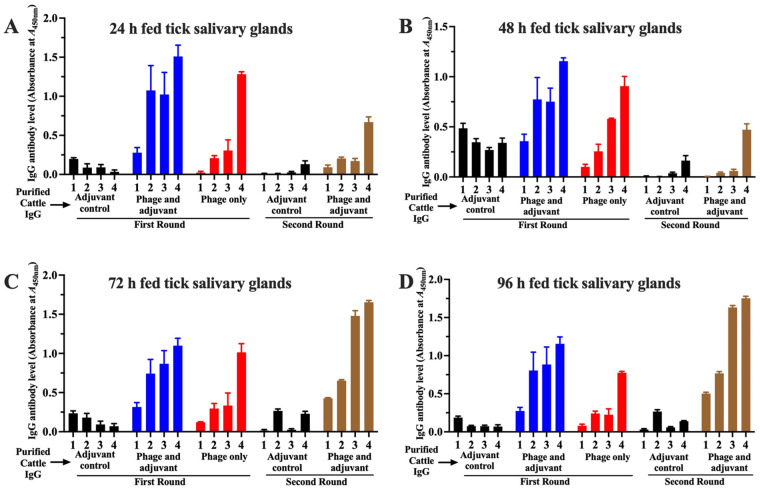
Cattle IgG antibodies to T7 phage-displayed *Amblyomma americanum* whole-tick antigens specifically react with native *A. americanum* salivary gland proteins. ELISA plates coated with whole salivary gland protein extracts from *A. americanum* ticks that were fed on cattle for 24 h (**A**), 48 h (**B**), 72 h (**C**), and 96 h (**D**) were screened with purified IgG from cattle sera at three weeks after priming dose (bar 1); three weeks after booster dose (bar 2), before tick feeding (bar 3), and at one day after tick feeding (bar 4). ELISA screening included sera from control cattle (SM buffer + adjuvant, black bars), T7 phage display library + adjuvant (blue bars), and T7 phage only (red bars) from first round immunization. The second round included adjuvant control (black bars) and T7 phage display library + adjuvant (brown bars). The *Y*-axis indicates optical density (OD) values at A450nm, with bars showing mean IgG binding levels ± SEM. Statistical significance between the immunized and control groups was determined using Student’s *t*-test.

We next assessed whether cattle antibodies to T7 phage-displayed *antigens* could recognize native tick salivary gland proteins. Consistently, cattle antibodies to T7 phage-displayed *antigens* specifically reacted with native *A. americanum* tick proteins that were extracted from salivary glands of ticks that were fed on cattle for 24, 48, 72, and 96 h (Figure 3A–D). Consistent with the elevated IgG responses observed against T7 phage-displayed antigens (Figure 2B), cattle immunized with adjuvant-formulated T7 phage-displayed tick antigens also generated strong IgG reactivity against native salivary gland proteins. Elevated IgG levels were further observed in all immunized groups post tick feeding, demonstrating a robust recall memory response (Figure 2 and Figure 3).

### 3.2. Cattle Immunized with T7 Phage-Displayed Whole-Tick Antigens Induce Antibodies Against Recombinantly Expressed Tick Saliva Proteins

**Figure 4:**
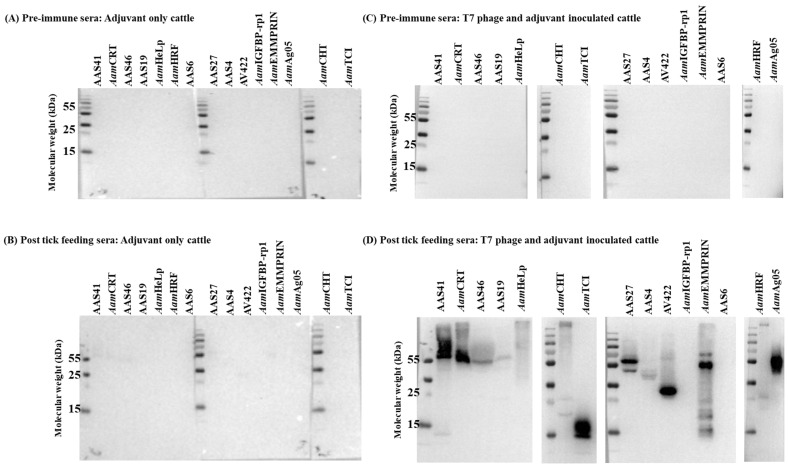
Cattle IgG antibodies to T7 phage-displayed *Amblyomma americanum* whole-tick antigens specifically react with recombinant *A. americanum* saliva proteins. Yeast-expressed recombinant *A. americanum* saliva proteins (Table 1) were resolved by SDS-PAGE (12%) and subjected to standard Western blotting assay as outlined in materials and methods. Membranes were probed with purified cattle IgG (25 µg/mL). Following incubation with HRP-conjugated chicken anti-bovine IgG (1:5000), blots were developed using HRP substrate and visualized with a ChemiDoc imaging system. Panels (**A**,**B**) were probed with pre-immune and post-infected purified IgG from the cattle immunized with adjuvant only. Panels (**C**,**D**) were probed with pre-immune and post-infected purified IgG from the cattle immunized with T7 phage-displayed library with adjuvant, respectively. AAS4, AAS6, AAS19, AAS27, AAS41, and AAS46 denote *A. americanum* tick serine protease inhibitors (serpins) 4, 6, 19, 27, 41, and 46, respectively. *Aam*HRF = *A. americanum* histamine release factor, *Aam*EMMPRIN = *A. americanum* extracellular matrix metalloprotease inducer, *Aam*CHT = *A. americanum* chitinase, MAC25 = *A. americanum* insulin-like growth factor binding protein related protein 1, *Aam*CRT = *A. americanum* calreticulin, HeLp = *A. americanum* heme lipoprotein, and *Aam*TCI = *A. americanum* tick carboxypeptidase inhibitor. AV422 and *Aam*Ag05 denote cross-tick species conserved *A. americanum* tick saliva proteins of unknown function. Distinct bands in panel (**D**) indicate specific reactivity of purified IgG to the indicated recombinant *A. americanum* tick saliva proteins.

**Figure 5:**
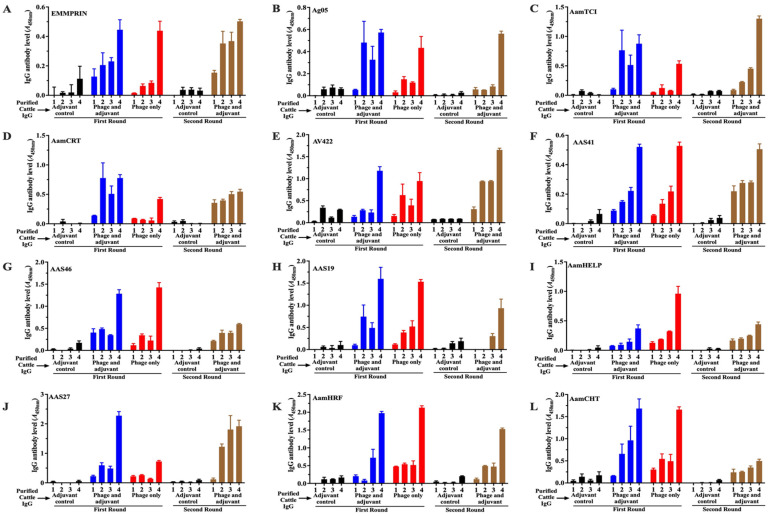
Cattle IgG antibodies to T7 phage-displayed *Amblyomma americanum* whole-tick antigens elicit variable IgG antibody levels to recombinant *A. americanum* tick saliva proteins. Affinity-purified recombinant tick saliva proteins (100 ng/well) were subjected to standard ELISA. ELISA screening included sera from control cattle (SM buffer + adjuvant, black bars), T7 phage display library + adjuvant (blue bars), and T7 phage only (red bars) from the first round of immunization. The second round included adjuvant control (black bars) and T7 phage display library + adjuvant (brown bars). IgG was purified from cattle sera at three weeks after the priming dose (bar 1); three weeks after the booster dose (bar 2), before tick feeding (bar 3), and at one day after tick feeding (bar 4). The recombinant proteins that were screened included (**A**) *Aam*EMMPRIN, (**B**) *Aam*Ag05, (**C**) *Aam*TCI, (**D**) *Aam*CRT, (**E**) AV422, (**F**) AAS41, (**G**) AAS46, (**H**) AAS19, (**I**) *Aam*HeLp, (**J**) AAS27, (**K**) *Aam*HRF, and (**L**) *Aam*CHT. The *Y*-axis indicates optical density (OD) values at A450nm, with bars showing mean IgG binding levels ± SEM. Statistical significance between treatment and control groups was determined using Student’s *t*-test.

Western blotting (Figure 4) and ELISA (Figure 5, Supplementary File S1) analyses of recombinantly expressed *A. americanum* tick saliva proteins confirmed that cattle antibodies to *A. americanum* proteins displayed on T7 phage specifically recognized tick proteins injected into cattle during tick feeding. We evaluated 15 recombinant tick saliva proteins (Table 1 and Supplementary Figure S1) that were previously identified from the T7 phage display library used for cattle immunization [15] and are known to be secreted during tick feeding [24]. Dot blot analysis using purified IgG from immunized cattle detected 13 of the 15 recombinant proteins (Figure 4 and Figure S2). Here, we used IgG that was purified from sera of immunized cattle following tick infestation (Supplementary Figure S3). Notably, IgG purified from pre-immune sera of both control and phage-immunized cattle showed no reactivity to the recombinant antigens, and IgG from control cattle post-tick feeding showed minimal immune response to the recombinant antigens (Figure 4A–C), demonstrating specificity. Notably, IgG purified from immunized cattle strongly reacted with *A. americanum* saliva serine protease inhibitors (AAS4, AAS19, AAS27, AAS41, and AAS46), calreticulin (*Aam*CRT), heme lipoprotein (*Aam*HeLp), AV422 antigen, *Aam*Ag05, chitinase (*Aam*CHT), histamine release factor (*Aam*HRF), extracellular matrix metalloprotease inducer (*Aam*EMMPRIN), and carboxypeptidase inhibitor (*Aam*TCI), all of which have been confirmed to be injected into the host during tick feeding (Figure 4D) [15,24]. These findings confirm that the observed antigen-specific IgG antibody response was induced through immunization of cattle with T7 phage-displayed *A. americanum* tick antigens.

We next determined the antibody levels to defined recombinant tick saliva proteins by ELISA (Figure 5, Supplementary File S1). This analysis revealed variable immunogenicity among the recombinant tick saliva proteins. Six of the antigens (AAS41, AAS46, AV422, *Aam*HRF, *Aam*EMMPRIN, and *Aam*CHT) exhibited similar IgG levels between cattle inoculated with adjuvant and non-adjuvanted vaccine formulations. Significantly lower IgG levels were observed in the adjuvant formulation group for *Aam*HeLp, while *Aam*TCI, *Aam*Ag05, *Aam*CRT, and AAS27 elicited stronger antibody responses in the formulation without adjuvant. It is notable that, in round 2 immunization, IgG levels to *Aam*CHT, *Aam*HRF, *Aam*HeLp, AAS46, and *Aam*CRT decreased significantly, while responses to the remaining proteins remained unchanged. Notably, antibody levels increased following tick feeding across all screens, indicating an anamnestic response in immunized cattle, suggesting immune memory.

### 3.3. Cattle Immunity to T7 Phage-Displayed Whole-Tick Antigens Reduces Tick Feeding Efficiency and Prevents Larval Tick Hatching

**Figure 6:**
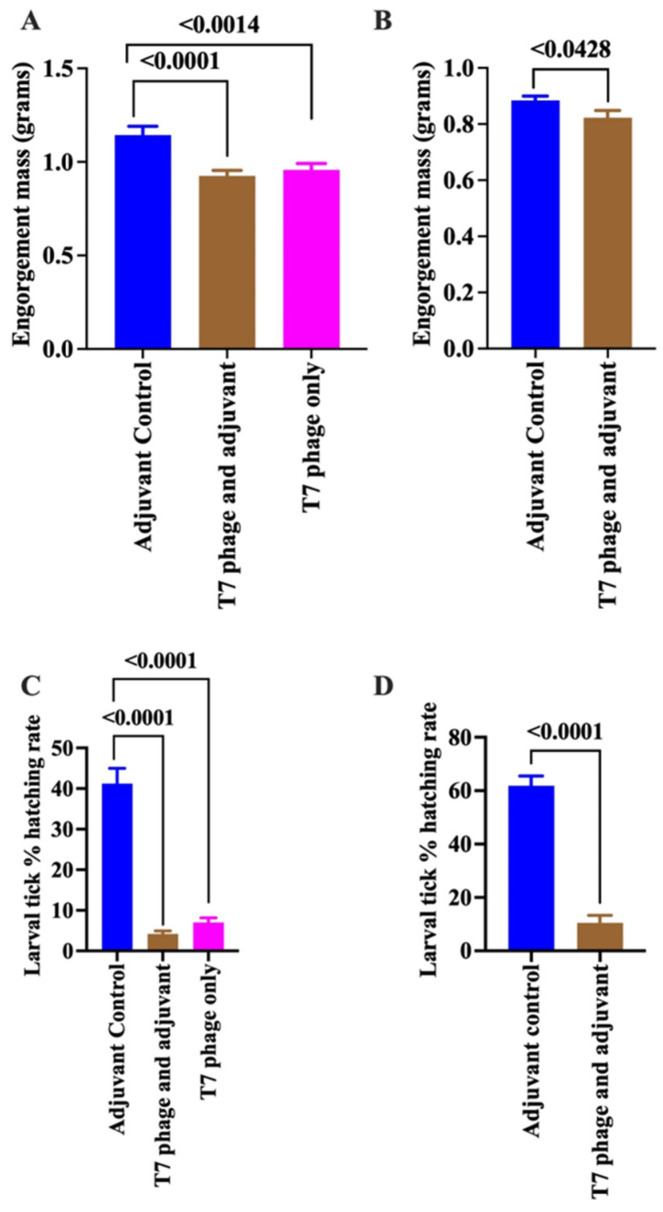
Immunization of cattle with T7 phage-displayed *Amblyomma americanum* whole-tick antigens confers protective immunity against tick feeding and inhibits larval hatching. Female *A. americanum* ticks were fed on control (adjuvant-inoculated) and cattle immunized with T7 phage, displaying *Amblyomma americanum* whole-tick antigens formulated without or with adjuvant, as indicated. Engorgement mass as an index for ingested blood (**A**,**B**) and larval hatching rate (**C**,**D**) were evaluated in two independent cattle immunization trials: Round 1 (**A**,**C**) and Round 2 (**B**,**D**). Blue bars denote ticks fed on adjuvant control cattle, and brown bars denote ticks fed on cattle immunized with adjuvanted T7 phage-displayed antigens. Pink bars denote ticks fed on cattle immunized with the T7 phage-displayed antigens without adjuvant. Data show mean values ± SEM. Statistical significance between groups was determined using an unpaired *t*-test with Welch’s correction.

Immunization did not affect the ability of ticks to attach or initiate feeding, as the duration of feeding and time to repletion were comparable between control ticks and those that fed on immunized cattle. However, blood meal feeding efficiency was significantly impaired in ticks that fed on immunized cattle. Engorgement weights, an index of the amount of blood ingested, markedly decreased from 1.144 ± 0.047 g in control ticks to 0.9252 ± 0.0298 g (*p* < 0.0001) for ticks that fed on cattle immunized with the adjuvanted vaccine and 0.9576 ± 0.03407 g (*p* < 0.0014) for ticks that fed on cattle immunized with the non-adjuvanted formulation (Figure 6A). A similar trend was observed in the second trial, where the average engorgement mass declined from 0.8846 ± 0.01541 g in control ticks to 0.8231 ± 0.02544 g (*p* < 0.0428) in ticks that fed on adjuvanted vaccine immunized cattle (Figure 6B). Although not statistically significant, ticks that fed on immunized cattle laid smaller egg masses, and the conversion efficiency of blood meal to eggs was unaffected. Significantly, the eggs laid by these ticks were largely non-viable, with only 4–7.5% hatching into larvae (Figure 6C,D).

## 4. Discussion

This study advances anti-tick vaccine development by advocating a novel multi-antigen delivery strategy utilizing the T7 phage display system. The concept of anti-tick immunization as an alternative to acaricide-based control stems from evidence that repeated exposure to tick saliva proteins in naturally infested animals induces strong immunity that disrupts tick feeding and prevents pathogen transmission [27,28]. Similar protective effects have been achieved using whole protein extracts from tick tissues [29,30]. However, except for the Bm86 vaccine developed against *R. microplus* [11], most single-antigen vaccines have shown limited efficacy [30]. To address this challenge, we evaluated the T7 phage display system as a platform to deliver a cocktail of *A. americanum* saliva protein antigens, aiming to mimic the broad, multi-antigen immune responses observed in repeatedly infested cattle that acquire potent anti-tick immunity [31]. Our data confirms that the T7 phage display system could be utilized to deliver multiple tick saliva antigens, which could adversely affect the ability of ticks to complete their life cycle.

This study builds upon previous research demonstrating the potential of bacteriophages as platforms for vaccine antigen delivery [32,33]. In the context of tick vaccine development, the M13 bacteriophage has been validated as a delivery vehicle for a peptide derived from the *R. microplus* midgut protein Bm86 (Sbm7462 antigen), successfully eliciting both humoral and cellular immune responses [34]. Complementary to this, our lab and others have employed phage immuno-screening systems to identify promising tick vaccine antigens [35]. Among the various phage systems available, we selected the T7 phage display system due to its distinct advantages, including rapid replication and efficient display of foreign antigens on its capsid surface [19]. These features make T7 a compelling candidate for scalable and targeted vaccine development.

A key concern in phage-based vaccine strategies is the potential for the host immune response to be directed predominantly against the phage carrier proteins rather than the displayed antigens, which could compromise vaccine efficacy. Notably, our findings indicate that immunization with T7 phages displaying tick-derived antigens elicits a robust and specific immune response primarily targeting the tick proteins, rather than the phage structural components. This highlights the potential of the T7 phage display system as a focused and effective platform for vaccine delivery. Our ELISA data further demonstrated that immunization with T7 phage-displayed tick proteins in cattle induced a canonical immune response, characterized by an early IgM response followed by class switching to IgG [36]. IgM typically represents the initial antibody response, indicating activation of naïve B cells by the vaccine antigens [36]. This suggests that the T7-displayed antigens were appropriately presented to the host immune system. Similar immune profiles have been reported in previous studies utilizing phage-delivered vaccine antigens [37,38]. Furthermore, the elevation of the class switching leading to elevated IgG levels could lead to the effective activation of adaptive immunity, the development of long-term, high-affinity antibodies, and the establishment of immune memory [36]. It is clearly evident that the *A. americanum* immunogens on the coat of T7 phages worked efficiently to elicit the immune response.

Another critical aspect of our study was evaluating whether the immune response elicited by T7-displayed antigens could recognize native tick saliva proteins. A known limitation of recombinant antigen approaches is the potential for epitope mismatches between the displayed and native proteins [39,40]. However, our data showed a strong antibody response to native tick salivary gland protein extracts, indicating that key antigenic epitopes were preserved in T7 phage-displayed tick saliva antigens. The specific reactivity of cattle immune sera to defined recombinant proteins in ELISA and Western blot assays further confirmed that the T7-displayed tick antigens were correctly presented and immunologically relevant.

Our laboratory and others have shown that diverse tick salivary proteins mediate robust anti-tick immunity acquired through repeated infestations in cattle and other animals. In this study, 13 out of 15 tested recombinant tick salivary proteins showed reactivity to immune sera from cattle, reinforcing the potential of the T7 phage system to deliver a multivalent, cocktail-based tick vaccine. A critical benchmark for successful immunization is the induction of durable immune memory, wherein re-exposure to the same antigen triggers a heightened immune response [41,42]. Notably, we observed elevated antibody titers following tick feeding on immunized cattle, providing strong evidence that T7 phage-displayed tick antigens elicited effective immune memory. The enhanced antibody response upon tick challenge, surpassing levels observed after booster immunization, suggests that native tick salivary proteins introduced during feeding were recognized by memory B cells primed by the T7 phage-displayed tick antigens, resulting in a rapid and amplified immune reaction.

The primary goal of anti-tick immunization is to disrupt tick feeding and reproduction, thereby preventing the transmission of tick-borne pathogens and reducing the sustainability of tick populations in the environment [43]. Studies conducted in the 1970s and 1980s demonstrated that immunizing cattle and other animals with whole-tick or tick organ protein extracts primarily impaired tick feeding and reproductive efficiency [44,45,46,47]. In these studies, cattle and other hosts immunized with protein extracts derived from whole ticks or specific internal organs, such as the gut, exhibited reduced blood meal feeding and, importantly, diminished reproductive success. Our findings here are consistent with these earlier observations. Immunization of cattle with a cocktail of *A. americanum* tick proteins displayed on T7 phage significantly reduced blood meal feeding efficiency and reproductive success. Ticks that fed on immunized cattle ingested smaller blood meals, laid fewer eggs, and, importantly, produced largely non-viable eggs, with hatching failure exceeding 95% across treatment groups. This pronounced reduction in egg viability highlights the potential of T7 phage-based immunization to effectively deliver a multivalent cocktail of anti-tick antigens, achieving protective outcomes comparable to those observed with whole-tick protein immunization [46]. The T7 phage-display system offers a feasible and practical approach to immunize cattle with a library of whole tick antigens cloned into the T7 phage display system.

The near-complete failure of egg hatching observed in this study is highly significant. Several important tick-borne pathogens are transovarially transmitted, including Heartland virus, *Rickettsia rickettsii*, responsible for Rocky Mountain spotted fever (RMSF) and Brazilian spotted fever across the USA, and notably *Borrelia lonestari*, a spirochete suspected to cause Southern tick-associated illness (STARI), a Lyme-like disease [48,49,50,51]. Similarly, this approach can also be tested on other pathogens known for transovarial transmission, such as *Babesia bovis* and *Babesia bigemina*, the causative agents of bovine babesiosis, which is a disease of major global significance [52,53]. These protozoan parasites are passed from infected female ticks to their progeny, enabling the infection of cattle through larval feeding [54,55]. Collectively, these pathogens are leading causes of human and livestock illness, and in many cases, their prevalence is amplified through efficient vertical transmission [56,57,58,59]. Therefore, preventing successful reproduction and larval emergence, through vaccination with T7 phage-displayed *A. americanum* whole-tick antigens, could reduce the transmission cycle of these pathogens. A vaccine capable of preventing larval tick hatching would provide a robust, sustainable strategy to disrupt the transovarial transmission and strengthen integrated control programs for ticks and tick-borne diseases.

It is essential to acknowledge the limitations of this study. First, we did not perform a dose optimization analysis to determine the most effective concentration of T7 phage displaying *A. americanum* whole-tick antigens. In this study, cattle were immunized with a fixed dose of 1.9 × 10^14^ plaque-forming units (PFU) of T7 phage particles. While this dosage elicited a robust immune response, it remains unclear whether it represents the optimal immunizing dose. Future studies are needed to establish the dose–response relationship and identify the minimal effective PFU required for protective immunity. Another limitation lies in the origin of the T7 phage display library, which was synthesized from cDNA derived from ticks that had fed on rabbits [15]. Previous work from our lab and others has shown that *A. americanum* and other tick species exhibit host-dependent gene and protein expression profiles during feeding [24]. This host-specific expression may explain why, despite immunization with multiple salivary proteins, tick attachment and initiation of feeding were not prevented. Additionally, the T7 phage display library used in this study was constructed from ticks collected at 24 and 96 h post-attachment [15]. Our prior research has demonstrated that tick salivary protein expression is highly dynamic throughout the feeding process [24,60], likely as an immune evasion strategy. Consequently, the immune response induced in this study may have been directed primarily against proteins expressed at those specific time points, potentially limiting its effectiveness against proteins secreted earlier or later during the feeding period. Despite these limitations, this study provides a practical framework for leveraging the T7 phage display system to deliver a multivalent vaccine composed of tick salivary proteins. This approach mimics the natural, broad-spectrum immunity observed in cattle repeatedly exposed to tick infestations, offering a promising strategy for future tick vaccine development.

## Figures and Tables

**Table 1:** Recombinant *A. americanum* tick saliva proteins screened in ELISA and Western blotting assays.

S. No.	Protein Nomenclature	Accession #	References
1.	AAS4	ABS87356.1	[26]
2.	AAS6	ABS87358.1	[26]
3.	AAS19	JAI08902.1	[26]
4.	AAS27	GAYW01000314.1	[26]
5.	AAS41	GAYW01000021	[26]
6.	AAS46	GAYW01000194	[26]
7.	*Aam*CRT	AAC79094.1	[15]
8.	*Aam*HeLp	ABK40086.2	[15]
9.	*Aam*AV422	AGH08176.1	[15]
10.	*Aam*TCI	KAK8762500.1	[15]
11.	*Aam*IGFBP-rp1	ADE06668.1	[15]
12.	*Aam*CHT	AIR95100.1	[15]
13.	*Aam*HRF	AAY67700.1	[15]
14.	*Aam*EMMPRIN	KAK8757866.1	[15]
15.	*Aam*Ag05	KAK8774087.1	[15]

## Data Availability

The original contributions presented in this study are included in the article/Supplementary Materials. Further inquiries can be directed to the corresponding author.

## Supplementary Materials

The following supporting information can be downloaded at: https://www.mdpi.com/article/10.3390/pathogens15030281/s1, Figure S1: Expression and purification of fifteen *Amblyomma americanum* salivary gland recombinant proteins. Recombinant proteins were expressed in *Pichia pastoris* strain X-33 using the pPICZαA recombinant protein expression system as outlined. Protein expression was induced in Buffered Methanol-complex Medium (BMMY), with methanol supplementation every 24 h. Optimal expression time points were determined by pilot-scale expression and subsequently scaled up to 1 L cultures. Secreted recombinant proteins were precipitated from culture supernatants by ammonium sulfate saturation, dialyzed, and affinity-purified using a HiTrap Chelating HP column. Purified proteins were analyzed by 12% SDS-PAGE followed by silver staining, confirming the high purity of all fifteen *A. americanum* recombinant immunogens used in this study; Figure S2: Dot blot identification of immunogenic *Amblyomma americanum* salivary antigens recognized by vaccine-induced antibodies. Fifteen recombinant *A. americanum* proteins (100 ng each) were spotted onto activated PVDF membranes and probed with pre-immune and post-infested IgG (25 µg/mL) purified from the cattle sera immunized with the *A. americanum* T7 phage display antigen with adjuvant. Bound antibodies were detected using HRP-conjugated chicken anti-bovine IgG and visualized by chemiluminescence, identifying recombinant proteins recognized by vaccine-induced antibodies. Reactive dots indicate recombinant proteins corresponding to immunogenic antigens represented within the *A. americanum* phage display library; Figure S3: Purification of IgG from the cattle immunized with *Amblyomma americanum* T7 phage display library. Serum IgG was affinity-purified using Protein A/G Sepharose beads following clarification by centrifugation and incubated for 3 h with pre-equilibrated beads at room temperature. The beads were washed with PBS, and the bound IgG was eluted under acidic conditions and immediately neutralized, collected, and stored for downstream analyses. Purified IgG was evaluated for purity by running 12% SDS-PAGE gel followed by Coomassie Blue staining (A–C, G–J) and by Western blotting (D–F, K–N). Samples shown are from control cattle (A, D, G, H, K, L), cattle immunized with the phage display library plus adjuvant (B, E, I, J, M, N), and cattle immunized with the phage display library without adjuvant (C, F), confirming successful isolation of high-purity IgG used in immunological assays; File S1: ELISA detection of antibody responses to *Amblyomma americanum* T7 phage-displayed whole-tick antigens, native salivary proteins, and recombinant proteins. ELISA plates were coated with purified *A. americanum* T7 phage display library antigens, native tick salivary gland protein extracts, or affinity-purified recombinant *A. americanum* salivary proteins. Following blocking, plates were incubated with cattle serum for the detection of IgM or IgG antibodies, or with affinity-purified bovine IgG, as indicated. Bound antibodies were detected using HRP-conjugated secondary antibodies and developed with TMB substrate, and absorbance was measured at 450 nm. The data shown represent raw ELISA absorbance values demonstrating antibody recognition of phage-displayed antigens, native salivary gland proteins, and recombinant tick salivary immunogens. Cattle serum IgM (Sheet 1) and IgG (Sheet 2) responses were measured against the whole *A. americanum* T7 phage display library. Affinity-purified IgG reactivity was assessed against native salivary gland extracts from ticks fed for 24 h (Sheet 3), 48 h (Sheet 4), 72 h (Sheet 5), and 96 h (Sheet 6), as well as against recombinant *A. americanum* salivary proteins: Ag05 (Sheet 7), AAS46 (Sheet 8), AAS41 (Sheet 9), AAS27 (Sheet 10), AaCRT (Sheet 11), AAS19 (Sheet 12), AaHELP (Sheet 13), AaCHT (Sheet 14), AaTCI (Sheet 15), AV422 (Sheet 16), EMMPRIN (Sheet 17), and AaHRF (Sheet 18).
