# Supplementary material for: Cattle Immunization with T7 Phage-Displayed Whole-Tick Antigens Reduces Amblyomma americanum Feeding Efficiency and Blocks Larval Tick Hatching
Source: Pathogens. 2026 Mar 5;15(3):281. doi: 10.3390/pathogens15030281 (PMC13028683; doi:10.3390/pathogens15030281)
Supplement: Supplementary file 1 [file pathogens-15-00281-s001.zip › Supplementary_Revised/Supplementary Figures_SF1-SF3_Revised.pptx]

## Slide 1
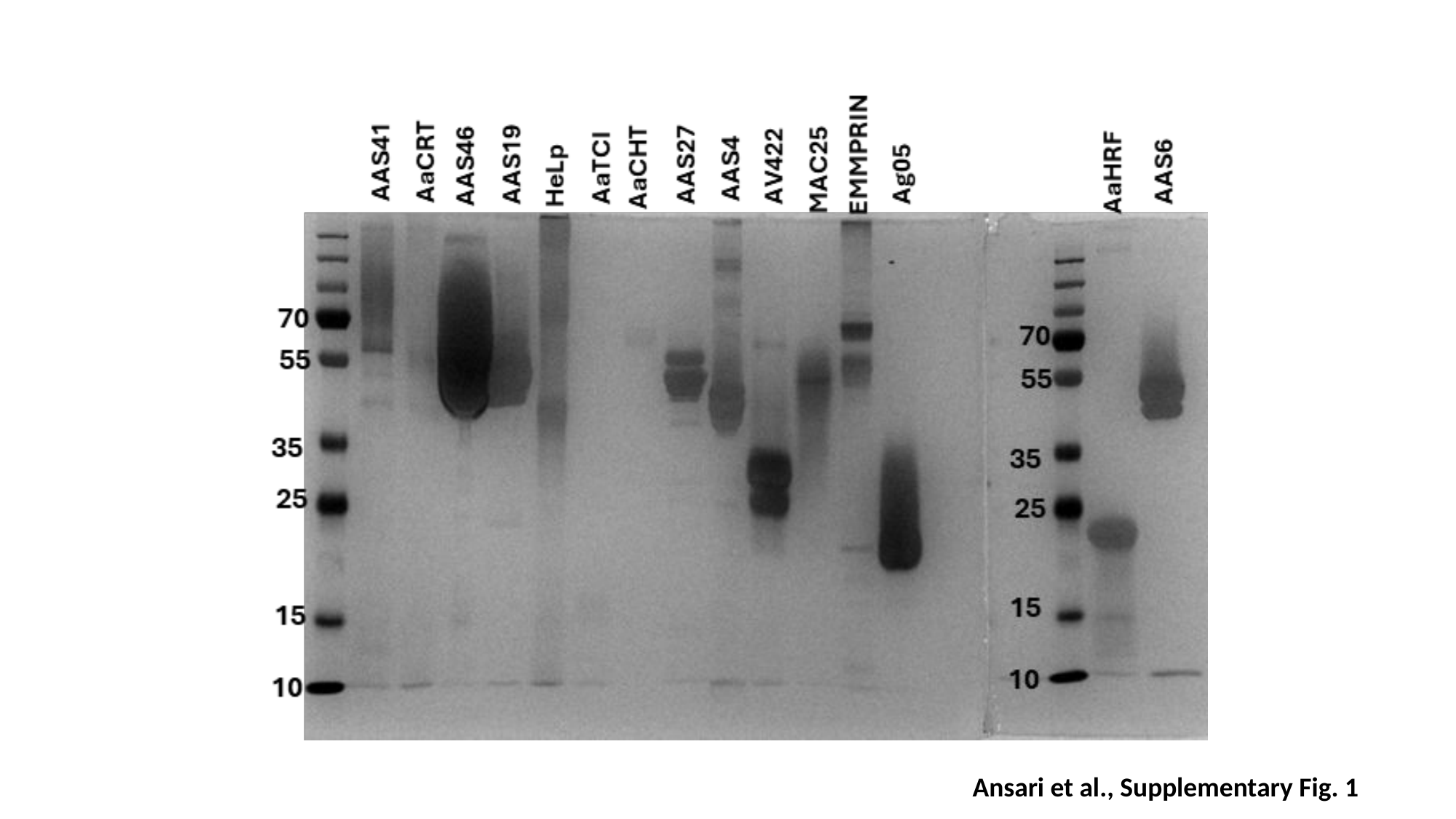

Ansari et al., Supplementary Fig. 1

## Slide 2
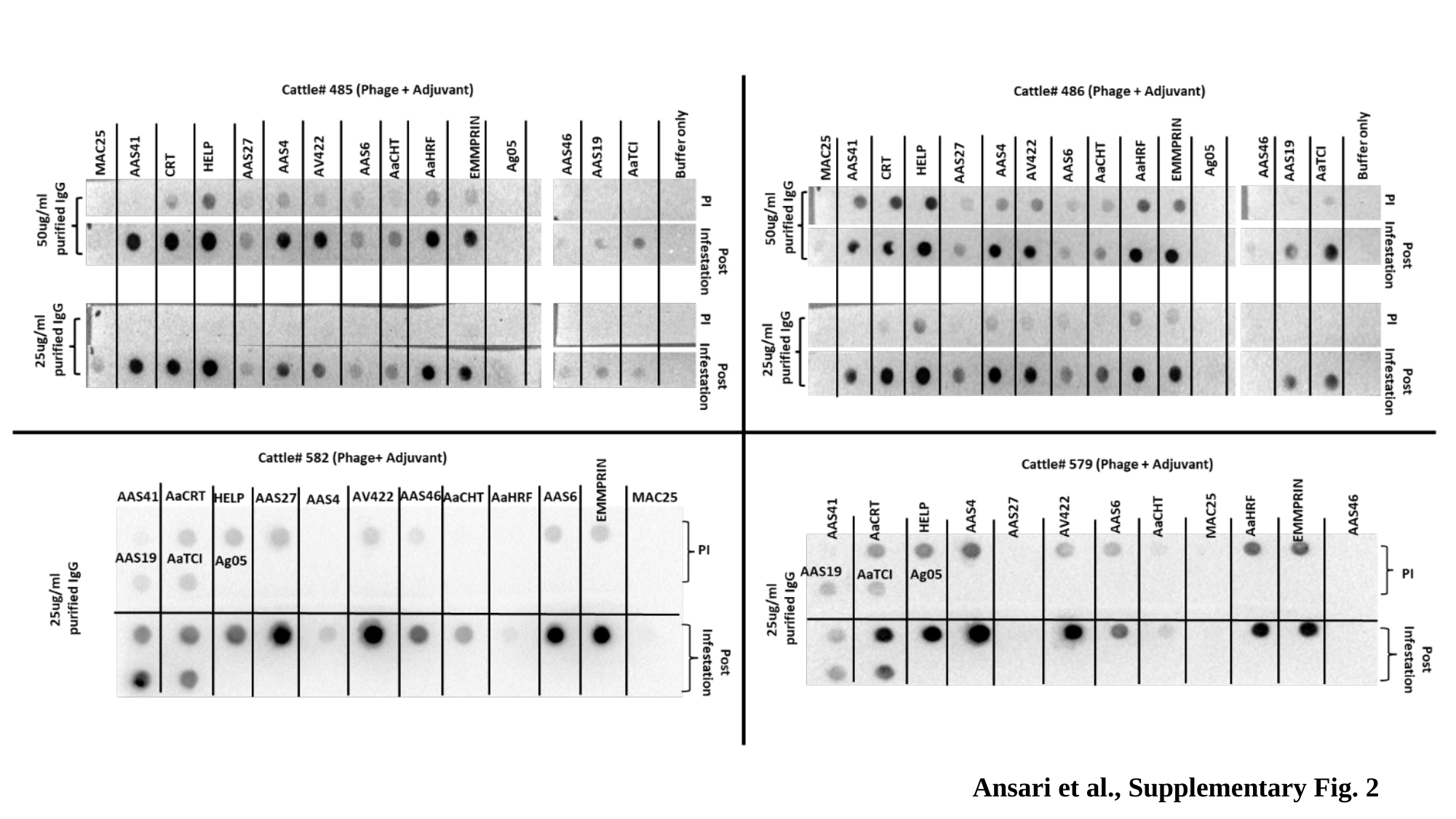

Ansari et al., Supplementary Fig. 2

## Slide 3
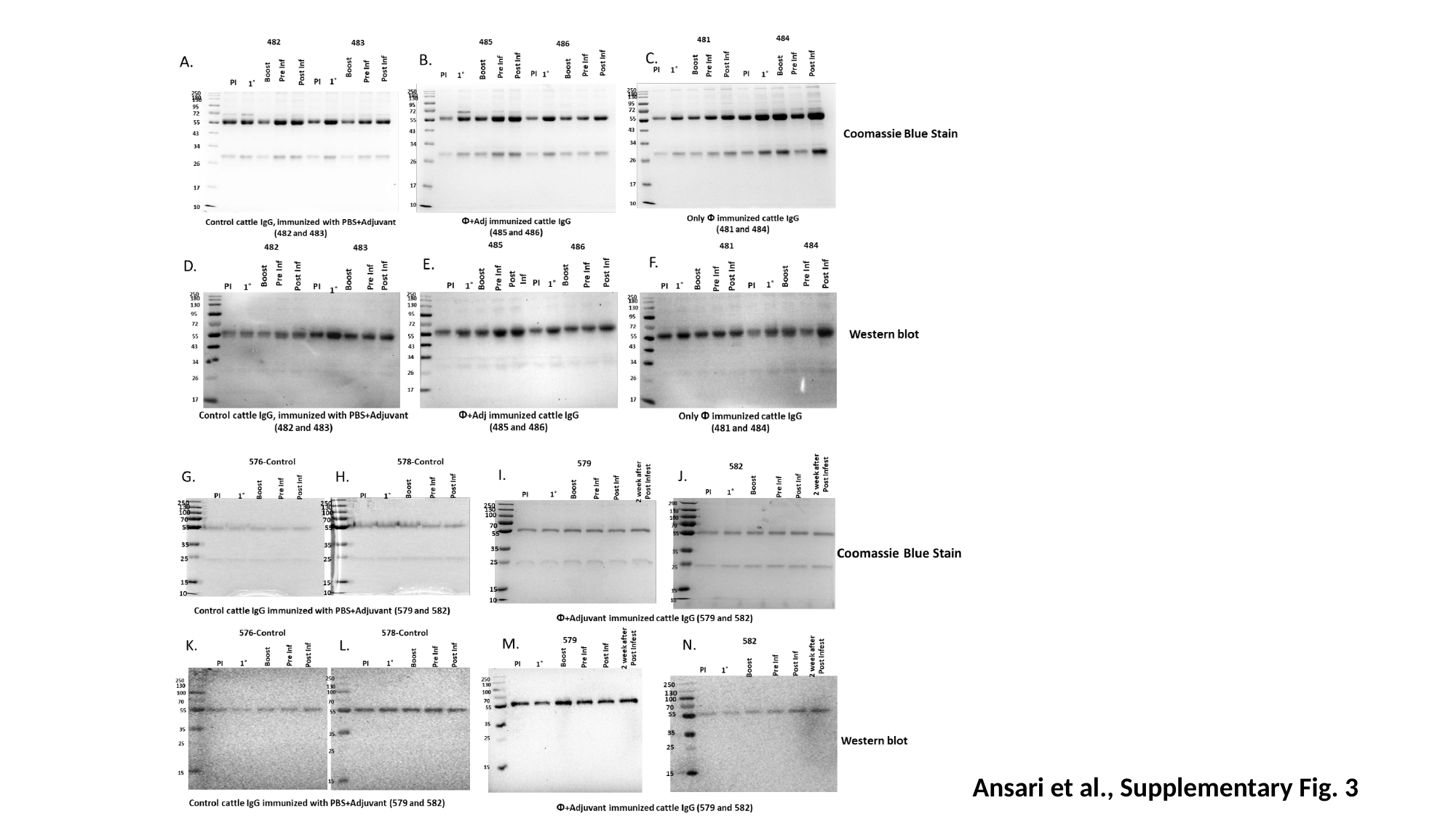

Ansari et al., Supplementary Fig. 3
